# Automatic Extraction of the Centerline of Corpus Callosum from Segmented Mid-Sagittal MR Images

**DOI:** 10.1155/2018/4014213

**Published:** 2018-07-04

**Authors:** Wenpeng Gao, Xiaoguang Chen, Yili Fu, Minwei Zhu

**Affiliations:** ^1^School of Life Science and Technology, Harbin Institute of Technology, Harbin, China; ^2^State Key Laboratory of Robotics and System, Harbin Institute of Technology, Harbin, China; ^3^Department of Neurosurgery, The Third People Hospital of Hainan Province, Sanya 572000, China; ^4^Department of Neurosurgery, The First Affiliated Hospital of Harbin Medical University, Harbin 150001, China

## Abstract

The centerline, as a simple and compact representation of object shape, has been used to analyze variations of the human callosal shape. However, automatic extraction of the callosal centerline remains a sophisticated problem. In this paper, we propose a method of automatic extraction of the callosal centerline from segmented mid-sagittal magnetic resonance (MR) images. A model-based point matching method is introduced to localize the anterior and posterior endpoints of the centerline. The model of the endpoint is constructed with a statistical descriptor of the shape context. Active contour modeling is adopted to drive the curve with the fixed endpoints to approximate the centerline using the gradient of the distance map of the segmented corpus callosum. Experiments with 80 segmented mid-sagittal MR images were performed. The proposed method is compared with a skeletonization method and an interactive method in terms of recovery error and reproducibility. Results indicate that the proposed method outperforms skeletonization and is comparable with and sometimes better than the interactive method.

## 1. Introduction

The corpus callosum (CC) is the main commissural bundle of fibers interconnecting the left and right cerebral hemispheres [1]. It facilitates interhemispheric communication in the human brain. Its special role has motivated imaging-based study of its size and shape to investigate the morphological correlation with various disorders, such as spastic cerebral palsy [2], fetal alcohol syndrome and fetal alcohol spectrum disorders [3], autism [4, 5], Turner syndrome [6, 7], HIV/AIDS [8], frontonasal dysplasia [9], dyslexia [10], attention-deficit hyperactivity disorder (ADHD) [11], and Alzheimer's disease [12, 13]. Most of these studies are based on the measurement of the CC's simplex geometric properties, such as the area [2, 6, 7, 12, 13] and circumference [11] of CC region, the angle between the CC and anterior-posterior commissure line [9]. However, these studies can only reveal the growth or atrophy of the entire CC but not exactly where the change occurs.

Recently, some researchers have focused on centerline-based analysis [3–5, 10], which is more powerful and comprehensive: centerline-based analysis can detect the exact position where the variation of thickness and angular change of the CC along the centerline occurs, which is more sensitive and discriminative in comparison with size- or area-based analysis. Nevertheless, the centerline is an implicit representation of the shape, and it is impossible to delineate the callosal centerline from magnetic resonance (MR) images manually. Many researchers have utilized skeletonization techniques to extract the skeleton as the main body of the centerline. The skeleton is a thin version of a shape, which is an important feature for shape description in image processing and computer vision. It is defined as the locus of centers of maximal inscribed disks in two dimensions (2D) [14]. In the technical literature, the concepts of skeleton and centerline are used interchangeably by some researchers, while others regard them as related, but not the same. In the view of anatomists, the centerline of the anatomy is not consistent with the skeleton, because the topology of the skeleton is uncertain, whereas the topology of the anatomical centerline is known. In general, the centerline starts and ends at boundary points. Taking the CC as an example, its centerline should be a curve that starts at the anterior pole of the rostrum and ends at the posterior pole of the splenium. Therefore, centerline extraction cannot completely depend on the techniques of skeletonization. However, the idea can be applied to extraction of the callosal centerline. Most previous centerline-based studies have adopted skeletonization techniques to extract the main part of the centerline and apply curve fitting after labeling the endpoints to obtain the centerline.

To date, diversified approaches have been proposed to extract the skeleton from an image. These approaches can be mainly classified into three categories: distance transformation based [15–19], Voronoi diagram based [20], and thinning based [21]. The distance transform computes the minimum distance of each pixel to the shape boundary. However, the distance transform is very sensitive to small perturbations of the boundary, as each value of the shape is assigned according to a single boundary point (the nearest point). The skeletons obtained by the distance transform require a pruning stage if the boundary is noisy [22]. To overcome the limitations of the distance transform, several smooth medial functions have been introduced based on Newton's law [23], electrostatic field [24], and Poisson's equation [25]. These methods consider several boundary points and therefore better reflect the global properties of the shape than does the distance transform. Thinning based methods involve a morphological operation that is used to remove object boundary pixels from binary images iteratively with a set of conditions, somewhat like erosion or gradual opening. Complex conditions are required to terminate this process and to preserve the topology and connectivity of the skeleton. In the Voronoi diagram-based approaches, the skeleton is extracted from a Voronoi diagram derived from the object boundaries. Existing skeletonization techniques suffer from at least one of the following shortcomings: dependence on the accuracy of determining the medial axis, computational complexity, lack of robustness, connectivity, spurious branches, or sensitivity to boundary noise. Therefore, the centerline cannot be precisely obtained through skeletonization alone.

**Figure 1:**
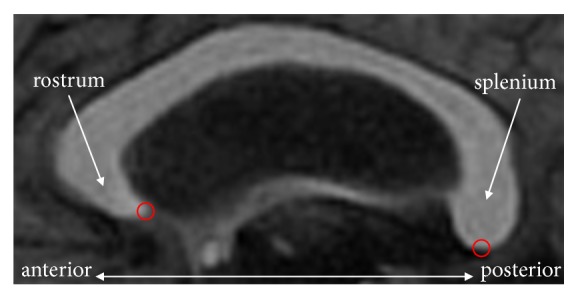
Endpoints (centers of the red circles) of the CC in a mid-sagittal MR image.

Localization of the anterior and posterior endpoints is another issue. The anterior and posterior endpoints are at the anterior and posterior poles of the CC, respectively, as shown in Figure 1. In general, the skeleton extraction methods cannot localize these two points, because they are not part of the skeleton according to the definition of a skeleton. The endpoint at the anterior pole of the rostrum (see Figure 1) is usually associated with a local maximum curvature of the callosal boundary [4, 5, 7]. Owing to the existence of noise on the boundary, it is not easy to locate it uniquely using state-of-the-art corner detection methods. As for the posterior endpoint, the problem is even more complicated, because the geometric features around it are not obvious, and there is no sharp tip in the splenium as exists in the rostrum. In addition, anatomical variability makes it more complicated to locate the posterior endpoint. Thompson et al. [8] selected the lowest points of the genu and splenium as the endpoints. In [11], the endpoints of the CC were determined by extending the centerline to the boundary. Owing to the inconsistent criteria for locating the endpoints, the results of these studies may also be inconsistent. To the best of our knowledge, there is no effective method that localizes these two endpoints.

Centerline-based shape analysis has been widely used in CC. Most research has adopted the method of skeletonization to extract the centerline. However, there are few works on the evaluation and validation of these centerline extraction techniques, which poses a rather serious challenge when interpreting their results. In this paper, we propose a method for the callosal centerline extraction from segmented mid-sagittal MR images. The main contributions of this paper are as follows. First, a method of model-based point detection is proposed to localize the callosal endpoints. A model for each endpoint is generated using statistical shape context as the descriptor under a local coordinate system, in which point detection is robust to boundary noise and is rotation invariant (to a certain extent). Then, active contour model (ACM) based curve evolution with two fixed endpoints is applied to approximate the centerline, which guarantees the topology of the obtained centerline and tolerates the influence of boundary noise. Experiments with 80 segmented mid-sagittal MR images were performed to evaluate the effectiveness of the proposed method.

## 2. Methods

### 2.1. Automatic Localization of the Endpoints

To automatically localize the two endpoints, a statistical model-based point detection method is proposed, which consists of two steps: model construction and point localization.

#### 2.1.1. Model Construction

**Figure 2:**
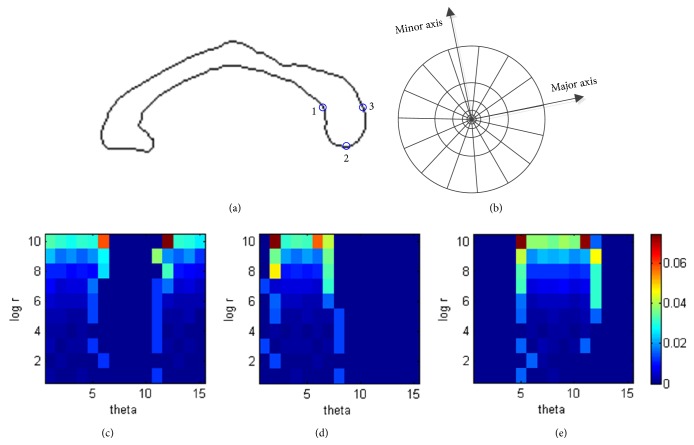
Shape context computation. (a) Contour of CC (black) and three boundary points marked by the centers of circles (blue). (b) Diagram of log-polar bins used in computing the shape contexts under the local log-polar coordinate system (10 bins for log⁡*r* coordinate and 15 bins for angular coordinate in this work). (c–e) Histogram maps of three points marked in (a).

The statistical models of the endpoints are generated using shape context [26], which is a robust, compact, and highly discriminative descriptor widely used in shape matching. The shape context of a point of interest is a measure of the distribution of other points in the shape relative to it under the log-polar coordinate system. Given a point *p*, the shape context of *p* is defined as a coarse histogram of the relative polar coordinates of the other points, written as

(1) $$\begin{array}{c} h\left(\;m,n\;\right)=\#\left\{\;q\neq p:\left(\;q-p\;\right)\in \mathrm{bin}\left(\;m,n\;\right)\;\right\} \end{array}$$

where *q* denotes the other points of the shape and *h*(*m*, *n*) is a normalized *M* × *N* bins histogram in log-polar space at *p*. Each bin indicates the proportion of the points in this region with respect to the total adjacent points of *p*.  Figure 2 illustrates the process of computing the shape context.

**Figure 3:**
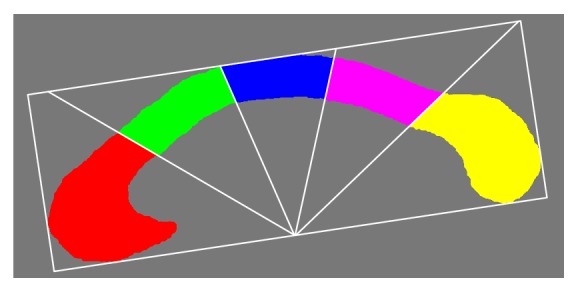
Subregions of CC. Red: rostrum and genu (CC1), green: rostral body (CC2), blue: mid-body (CC3), pink: isthmus (CC4), and yellow: splenium (CC5).

It is easy to make the shape context scale invariant, but we cannot guarantee rotation invariance by referring to the image coordinate system owing to different scanning directions and the existence of individual variability. Some methods obtain the shape context with respect to the tangent direction at the point, which may lose orientation information of the point and cause the shape context to be less sensitive when distinguishing similar boundary points. Alternatively, two local Cartesian coordinate systems with respect to the rostrum and splenium are defined for the computation of the two endpoints' shape contexts. Given a segmented CC, its bounding rectangle and major and minor axes are extracted using the method proposed by Chaudhuri and Samal [27]. Then, the CC is automatically divided into five subregions according to a modification of the Witelson partitioning scheme [28, 29]. Four radial dividers emanate from the midpoint of the inferior side of the bounding rectangle with equal angular interval and divide the CC into five subregions, i.e., the rostrum and genu (denoted as CC1), the rostral body (denoted as CC2), the mid-body (denoted as CC3), the isthmus (denoted as CC4), and the splenium (denoted as CC5) (see Figure 3). The log-polar coordinate system is defined on CC1 (and CC5) with its origin at the mass center of CC1 (and CC5) and radial axis parallel to the major axis of CC1 (and CC5) from anterior to posterior (see Figure 2(b)). The radial coordinate is divided by the height of the bounding rectangle of the CC for normalization, which guarantees scale invariance of the shape context.

Results may be biased if the endpoint with its shape context is derived from only one individual's CC. Therefore, we create a statistical model using the mean shape context as the descriptor. Suppose there are *K* samples (i.e., segmented CC images) in the training sets. Two raters are asked to label the endpoints by mutual agreement. Then, the shape context of each sample (denoted as *h*_*k*_) is calculated according to (1) and all shapes are aligned with respect to a local log-polar coordinate system. The mean shape context (denoted as $\overset{-}{h}$) is then written as

(2) $$\begin{array}{c} \overset{-}{h}\left(\;m,n\;\right)=\frac{1}{K}\overset{K}{\underset{k=1}{\sum }}h_{i}\left(\;m,n\;\right) \end{array}$$

Here, a statistical model is created and used to detect the endpoint by matching the model with the shape contexts of the candidate points.

#### 2.1.2. Point Localization

Given a segmented CC, *p*_*j*_ denotes a candidate point on the boundary. The process of locating the endpoints is to find a boundary point whose shape context is most similar to the model. As the shape context is represented as a histogram, the similarity is measured as the sum of the difference of two histograms according to

(3) $$\begin{array}{c} p^{*}=\underset{p_{j}}{\arg\;\min}\underset{m}{\sum }\underset{n}{\sum }\left|\;\overset{-}{h}\left(m,n\right)-h_{j}\left(m,n\right)\;\right| \end{array}$$

Here, *h*_*j*_(*m*, *n*) is the shape context of *p*_*j*_ and $\overset{-}{h}(m,n)$ is the statistical model obtained from (2).

### 2.2. Active Contour Based Centerline Extraction

The invisible CC centerline is approximated using the ACM proposed by Kass et al. [30]. The advantage of ACM is that the topology of the curve can be preserved during its evolution, which means that spurious branches can be avoided. To allow the curve to approximate the invisible centerline of the CC, the representation of the centerline should be introduced in advance.

#### 2.2.1. Representation of the Centerline

The centerline is depicted implicitly using a distance map [31], which labels each pixel with the distance to the nearest boundary pixel. If a pixel in the CC is labeled with a maximum distance, it means that this pixel is far from the boundary and in the center of the CC. Therefore, this pixel may be on the centerline. Let *O* denote the segmented CC region, and ∂*O* denote the boundary of *O*. We refer to *d*(*p*, *q*) as the Euclidean distance between two pixels *p* and *q*. The distance map in the CC is defined as

(4) $$\begin{array}{c} D_{O}\left(\;p\;\right)=\left\{\begin{array}{cc} \underset{\forall q\in \partial O}{\min}d\left(p,q\right) & p\in O\\ 0 & p\notin O \end{array}\right. \end{array}$$

#### 2.2.2. Evolution of the Curve

To fit the centerline using ACM, a curve **x**(*s*) = [*x*(*s*), *y*(*s*)], *s* ∈ [0,1] moves within the spatial domain of the distance map by minimizing the following energy function:

(5) $$\begin{array}{c} E=\overset{1}{\underset{0}{\int }}\frac{1}{2}\left(\;\alpha {\left|\;x^{\prime }\left(\;s\;\right)\;\right|}^{2}+\beta {\left|\;x"\left(\;s\;\right)\;\right|}^{2}+E_{ext}\left(\;x\left(\;s\;\right)\;\right)\;\right)ds \end{array}$$

where *α* and *β* are weighting parameters that control the curve's tension and rigidity, respectively, and **x**′(*s*) and **x**"(*s*) denote the first and second derivatives of **x**(*s*) with respect to *s*. The external energy *E*_*ext*_ is a function derived from the distance map of the CC and is responsible for driving the curve to the maximum distance region where the centerline is located. The formulation of *E*_*ext*_ is

(6) $$\begin{array}{c} E_{ext}=\frac{2}{\left(1-e^{\left|D\left(x\left(s\right)\right)\right|}\right)}\left|\;\nabla D\left(\;x\left(\;s\;\right)\;\right)\;\right| \end{array}$$

where *D* is the normalized distance map. The gradient flow ∇*D*(**x**(*s*)) moves the curve toward to the centerline. The coefficient 2/(1 − *e*^|*D*(**x**(*s*))|^) is used to modulate the force of the gradient flow of the distance map. When **x**(*s*) is near the boundary (or center) of the CC, the coefficient is close to 1.0 (or 0.0), and the external energy is increased (or decreased). This guarantees that the curve approximates the centerline more stably.

The model is initialized with a spline curve starting at the anterior endpoint, ending at the posterior endpoint, and passing through four control points in the CC. These four control points are on the four radial lines shown in Figure 3 (the maximum distance points). Then, a spline curve is interpolated with nearly equal distance intervals for initialization of the model. During the evolution of the curve, the endpoints of the curve are fixed at the detected endpoints.

## 3. Experiments and Results

The proposed method was implemented using the C++ language. The experiments were performed on an HP workstation with Intel Xeon CPU (E5540@ dual-core, 2.53 GHz) and 8 GB RAM. In the experiments, the weighted coefficients *α* and *β* in (5) were empirically set to 0.1 and 0.5, respectively.

To compare our method with existing methods, a skeletonization method and an interactive method were implemented. The skeletonization method (pfSkel-1.2.1.1) proposed by Chuang et al. [32] is publicly available (http://coewww.rutgers.edu/www2/vizlab/NicuCornea/Skeletanization/skeletanization.html).pfSkel mainly consists of four steps. First, a 2D vector field in the segmented CC is calculated with respect to the boundary pixels. Second, the critical points of the vector field are detected as the core skeleton. Third, the first level skeleton is generated from the divergence of the vector field. Last and fourth, the second level skeleton is derived by connecting the boundary pixel with a certain percentage of curvature value to the core and first level skeleton. The interactive method is based on the pfSkel method and consists of three steps. First, the skeleton is extracted from the segmented CC. Then, the anterior and posterior endpoints are labeled manually. Finally, the centerline is obtained with a cubic spline connecting the endpoints and fitting the skeleton. For clarity, pfSkel is denoted as SKEL1 (which only generates the first level skeleton) and SKEL2 (which generates the first level plus second level skeletons). The interactive method and our method are denoted as CLM and CLA, respectively.

### 3.1. Data and Preprocessing

The data sets for evaluating the presented method contain high-resolution T1-weighted MR brain volumes of 80 subjects, including 50 healthy controls and 30 patients with various pathologies (infarctions); subject ages range from 12 to 60 years. The volume size varies from 192×256×256 to 256×181×256 voxels. The voxel size ranges from 0.897 mm to 1 mm in the sagittal plane, from 0.879 mm to 1.25 mm in the coronal plane, and from 0.67 mm to 1.5 mm in the axial directions.

The CC in the mid-sagittal plane was segmented with our self-developed software applying the following steps: (1) resampling each volume to make it isotropic; (2) extracting the mid-sagittal MR image using the method proposed by Hu and Nowinski [33]; (3) Binarizing the mid-sagittal MR image with upper and lower thresholds determined using Gaussian mixture modeling [34]; (4) extracting the bounding rectangle of each region and calculating geometric parameters (such as length and width) using the method proposed by Chaudhrui and Samal [27]; (5) selecting the CC region according to its anatomic characteristics: (a) length (from the anterior point to posterior point) of 7 to 9 cm, (b) width (from the superior point to the inferior point) of 2 to 4 cm, (c) orientation (angle of the major axis with respect to the horizontal axis) from 5° to 40°, and (d) area > 2 cm^2^; and (6) manually rectifying any mis-segmentation or oversegmentation by two raters in mutual agreement. After the segmentation, the centerline endpoints were manually identified on the boundary of the CC by two experts according to their anatomical knowledge after mutual agreement.

### 3.2. Accuracy of Endpoint Localization

To validate the accuracy of the presented endpoint localization method, statistical models were generated with 15 samples in the datasets using the method described in Section 2.1.1. Then, the statistical models were used to localize the endpoints in the other 65 samples in the datasets. The endpoint localization error is measured as the distance between the detected point and the manually labeled point. The endpoint localization error was 0.85±0.12 mm in this case.

### 3.3. Qualitative (Visual) Evaluation

**Figure 4:**
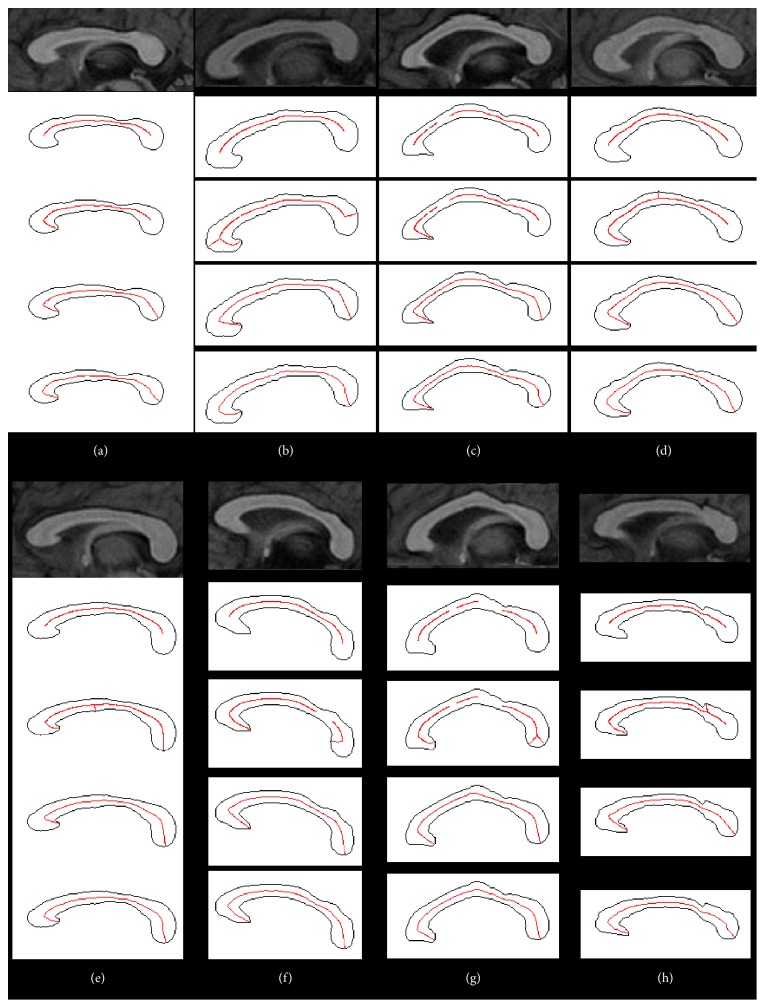
Results (red curves) for eight mid-sagittal MR images. Top row: mid-sagittal MR images, second row: results of SKEL1, third row: results of SKEL2, fourth row: results of CLM, and bottom row: results of CLA.

We present the results of SKEL1, SKEL2, CLM, and CLA to illustrate the difference in the centerline extraction in Figure 4. The top row illustrates the mid-sagittal MR images. Owing to the existence of intersubject variability, the shape of the CC varies significantly among the eight subjects. The results of SKEL1 are shown in the second row. The skeletons are not continuous and do not start and end at the anterior and posterior poles of the CC (see Figures 4(c) and 4(g)). The third row illustrates the results of SKEL2, in which the percentage was set to 0.001 experimentally. It generates fewer branches and more skeletons near the centerline. Even though the parameter has been adjusted to reduce the number of branches, there are still some spurious branches present (see Figures 4(b), 4(d), 4(e), 4(g), and 4(h)). The fourth row demonstrates the centerline extracted by an experienced and well-trained rater with the interactive method. The bottom row exhibits the results of the proposed method. The extracted centerlines are continuous curves connecting the anterior pole to the posterior pole and are centered in the region of the CC.

### 3.4. Quantitative Evaluation

The centerline is a geometric feature of a shape and is essentially invisible to the naked eye. Given a segmented CC, no radiologist or anatomist can manually delineate a centerline as the ground truth. Hence, it is difficult to validate the accuracy of the proposed method straightforwardly. In this paper, we adopted a technique used in assessing skeletonization results as proposed by Direkoglu et al. [35].

Suppose a point set *P* represents the extracted centerline. According to the definition of the centerline, a point *p* on the centerline must be the center of a maximum disk inscribed in the CC's shape. Let *r*_max_(*p*) denote the radius of the maximal disk *B*(*s*, *r*(*p*)) centered at the point *p*. The reconstruction of the CC region is given by

(7) $$\begin{array}{c} R\left(\;P\;\right)=\underset{p\in P}{⋃}B\left(\;s,r\left(\;p\;\right)\;\right) \end{array}$$

where *R*(*P*) is the reconstructed CC region. The quality of the extracted centerline is evaluated using a reconstruction error rate (RER) between the reconstructed and the segmented regions of the CC, which is calculated as follows:

(8) $$\begin{array}{c} RER=\frac{A\left(O\right)-A\left(R\left(P\right)\right)}{A\left(O\right)} \end{array}$$

where *A*(*∗*) is a function used to calculate the area measured in pixels. *O* and *R* represent the images that contain the segmented and reconstructed regions of the CC, respectively.

**Figure 5:**
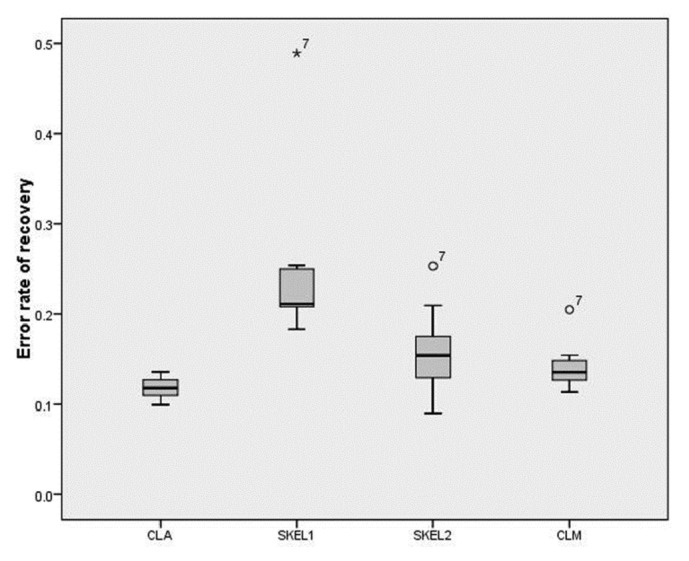
Boxplot chart of the RERs of SKEL1, SKEL2, CLA, and CLM.

We reconstructed the regions of the CC using the centerlines obtained by SKEL1, SKEL2, CLM, and CLA, respectively. RER is calculated using (8).  Figure 5 shows the RERs of SKEL1, SKEL2, CLM, and CLA. The mean and standard deviation of the RERs of CLA, SKEL1, SKEL2, and CLM are 0.12±0.01, 0.24±0.08, 0.15±0.04, and 0.14±0.02, respectively. The presented method outperforms the skeletonization methods (SKEL1 and SKEL2) and is comparable with or even better than CLM in terms of the RER.

### 3.5. Reproducibility Evaluation

To compare the presented method with CLM in terms of reproducibility, we randomly selected 10 samples in the dataset. For each sample, we extracted the centerline 10 times with the presented method and CLM, respectively. Five knowledgeable raters were asked to extract the centerline of each case with CLM once per day to guarantee that the raters were not influenced by previous results. Then, we obtained two groups of centerlines, i.e., one group with our method and another one with CLM. In each group, the distance between any two centerlines was calculated, and the mean distance was denoted as the reproducibility error.

Owing to the discretization of the centerline, the distance between two centerlines is measured as the distance between two point sets representing the centerlines. Given two centerline point sets *P* = {*p*_1_, *p*_2_,…, *p*_*M*_} and *Q* = {*q*_1_, *q*_2_,…, *q*_*N*_}, the mean distance between them using Euclidean distance is

(9) dP,Q=121M∑i=1Mminj=1,2,…,N⁡pi−qj+1N∑j=1Nmini=1,2,…,N⁡pi−qj

There was no reproducibility error with our method and an average reproducibility error of 0.047±0.003 mm with CLM.  Table 1 shows the results of CLM in detail.

## 4. Discussion

The corpus callosum plays an important role in the communication between the left and right cerebrums. Due to its essential role, its dysfunction may cause various neuropsychological or neuropathological diseases, while the progression of these diseases may also cause it to physically change shape and/or thickness. MRI-based morphology analysis of the CC has become an effective technique to investigate the variation of the CC in relation to these diseases in vivo. Most studies are based on area measurement [2, 13]. These methods can only detect the whole body change of the CC, which cannot describe the shape change in detail, and their findings are not sensitive or discriminative to specific diseases. To reduce this limitation, some researchers proposed measuring the area of the CC's subregions, which are obtained according to its geometric features [1, 12]. There are several rules to divide the CC [29, 36–38]. More recent studies based on these schemes have generated controversial results concerning the assumed topography of the callosal fiber tracts. Recently, several groups have focused on centerline-based analysis of the CC [3–5, 10], which is a promising way to investigate the variation of the CC in relation to specific diseases. The centerline, as a compact representation of the CC's shape, can be used to measure the thickness of the CC and the curvature at any centerline point; these descriptors provide comprehensive information regarding the CC's shape. In addition, the correspondence information among samples can be achieved easily, which facilitates population-based analysis, also known as centerline-based morphological analysis.

The presented method consists of two steps: automatic localization of the endpoints and ACM-based centerline extraction. There are three advantages to the presented method. First, the endpoint localization method is robust to boundary noise in comparison with methods based on curvature because the statistical shape context as a descriptor of local shape features can avoid the disturbances caused by noise. Second, the endpoints localization method is scale invariant due to normalization of the shape context. In addition, the endpoints localization method is rotation invariant to some extent. This is owing to the adoption of the local coordinate system, which makes our method robust to the rotation derived from not only the scanning direction but also individual variability in the CCs of different people. Despite the CC inclining forward and backward with respect to the horizontal line in Figures 4(b) and 4(f), our method can localize the endpoints accurately. The shape context is calculated with 15 bins in (1) (see Figure 2). In theory, the presented method can accommodate an angle between the model and the sample if less than 24°  ( = 360°/15). Figures 4(d) and 4(e) show the case for different angles between the rostrum and the body owing to the existence of intersubject variability. Finally, our method preserves topology by utilizing ACM to fit the centerline: a smooth centerline with no branches is obtained. In contrast, it is difficult to control the topology of the centerline using skeletonization. There is also no gap in the extracted centerline using our method, while gaps may exist in the skeletonization centerline (see Figures 4(c) and 4(g)).

The presented method has a lower RER in comparison with SKEL1, SKEL2, and CLM. This contributes to an automatic, accurate, and robust method for locating the endpoints and reproducing the centerline using ACM-based curve evolution. However, the recovery error cannot reach zero due to the irregular shape of any CC. In terms of reproducibility, the presented method has a higher accuracy in comparison with CLM because the endpoints localization method is more consistent in contrast to manual labeling by raters' subjective analysis.

The accuracy of the endpoint localization will affect that of the centerline extraction. However, the influence is limited owing to the movement of the curve in ACM mainly driven by the gradient flow of the distance map. The endpoint localization error merely interferes in the curve's behavior near the endpoints. The movement of the curve's main body is still under the supervision of the gradient flow. Moreover, the proposed method presents high accuracy (0.85±0.12mm) and robustness (see Figure 4) in the endpoint localization.

The prerequisite of the presented method is that the CC should be segmented from a mid-sagittal MR image in advance. To date, there are several techniques available to extract the mid-sagittal MR image automatically [33, 39–41] and various methods to delineate the corpus callosum, such as mathematical morphology-based methods [42], cluster-based methods [43, 44], deformable mode-based methods [45], tractography-based methods [46, 47], and template-based methods [48]. Any of these methods can be integrated into the presented method for convenience.

## 5. Conclusions

The centerline of the CC can depict the CC's shape variation in more detail when compared to size or area measurements. In this paper, we proposed a method of automatic extraction of the callosal centerline. The anterior and posterior endpoints are localized using statistical model-based point matching, which is robust to boundary noise and is rotation invariant to a certain extent. The centerline is fitted using the active contour model driven by a gradient of the distance map to produce an implicit representation of the centerline. Experiments with segmented MR images were performed to validate this method and the results indicate that our method outperforms skeletonization and is comparable with and sometimes better than the interactive method.

In the future, neurological or neuropathological diseases related to changes in the corpus callosum can be analyzed with centerline-based measurements, such as variation of thickness and curvature.

## Figures and Tables

**Table 1:** Reproducibility errors of CLM (unit: mm).

	Mean	SD.	Max	Min
Sample 1	0.012	0.003	0.034	0.002
Sample 2	0.023	0.009	0.067	0.001
Sample 3	0.071	0.010	0.106	0.015
Sample 4	0.044	0.009	0.082	0.009
Sample 5	0.042	0.007	0.067	0.014
Sample 6	0.072	0.014	0.139	0.021
Sample 7	0.119	0.015	0.214	0.039
Sample 8	0.072	0.089	0.113	0.025
Sample 9	0.063	0.009	0.106	0.021
Sample 10	0.066	0.008	0.103	0.030

## Data Availability

The data used to support the findings of this study are available from the corresponding author upon request.

## References

[1] Giedd J. N., Rumsey J. M., Castellanos F. X. (1996). A quantitative MRI study of the corpus callosum in children and adolescents. *Developmental Brain Research*.

[2] Kułak W., Sobaniec W., Kubas B., Walecki J. (2007). Corpus callosum size in children with spastic cerebral palsy: Relationship to clinical outcome. *Journal of Child Neurology*.

[3] Li L., Coles C. D., Lynch M. E., Hu X. (2009). Voxelwise and skeleton-based region of interest analysis of fetal alcohol syndrome and fetal alcohol spectrum disorders in young adults. *Human Brain Mapping*.

[4] Casanova M. F., El-Baz A., Elnakib A. (2011). Quantitative analysis of the shape of the corpus callosum in patients with autism and comparison individuals. *Autism*.

[5] El-Baz A., Elnakib A., Casanova M. F. (2011). Accurate automated detection of autism related corpus callosum abnormalities. *Journal of Medical Systems*.

[6] Fryer S. L., Kwon H., Eliez S., Reiss A. L. (2003). Corpus callosum and posterior fossa development in monozygotic females: A morphometric MRI study of Turner syndrome. *Developmental Medicine & Child Neurology*.

[7] Plessen K. J., Wentzel-Larsen T., Hugdahl K. (2004). Altered interhemispheric connectivity in individuals with Tourette's disorder. *The American Journal of Psychiatry*.

[8] Thompson P. M., Dutton R. A., Hayashi K. M. (2006). 3D mapping of ventricular and corpus callosum abnormalities in HIV/AIDS. *NeuroImage*.

[9] Giffoni S. D. A., Gimenes Gonçalves V. M., Zanardi V. A., Gil Da Silva Lopes V. L. (2004). Angular analysis of corpus callosum in 18 patients with frontonasal dysplasia. *Arquivos de Neuro-Psiquiatria*.

[10] Elnakib A., Casanova M. F., Gimelrfarb G., Switala A. E., El-Baz A. (2012). Dyslexia diagnostics by 3-D shape analysis of the corpus callosum. *IEEE Transactions on Information Technology in Biomedicine*.

[11] McNally M. A., Crocetti D., Mahone E. M., Denckla M. B., Suskauer S. J., Mostofsky S. H. (2010). Corpus callosum segment circumference is associated with response control in children with attention-deficit hyperactivity disorder (ADHD). *Journal of Child Neurology*.

[12] Zhu M., Gao W., Wang X., Shi C., Lin Z. (2012). Progression of Corpus Callosum Atrophy in Early Stage of Alzheimer's Disease. MRI Based Study. *Academic Radiology*.

[13] Zhu M., Wang X., Gao W. (2014). Corpus callosum atrophy and cognitive decline in early Alzheimer's disease: Longitudinal MRI study. *Dementia and Geriatric Cognitive Disorders*.

[14] Lieutier A. Any open bounded subset of ℝn has the same homotopy type than its Medial Axis.

[15] Arcelli C., Sanniti di Baja G. (1992). Ridge points in Euclidean distance maps. *Pattern Recognition Letters*.

[16] Kimmel R., Shaked D., Kiryati N., Bruckstein A. M. (1995). Skeletonization via Distance Maps and Level Sets. *Computer Vision and Image Understanding*.

[17] Malandain G., Fernández-Vidal S. (1998). Euclidean skeletons. *Image and Vision Computing*.

[18] Hesselink W. H., Roerdink J. B. T. M. (2008). Euclidean skeletons of digital image and volume data in linear time by the integer medial axis transform. *IEEE Transactions on Pattern Analysis and Machine Intelligence*.

[19] Ward A. D., Hamarneh G. (2010). The groupwise medial axis transform for fuzzy skeletonization and pruning. *IEEE Transactions on Pattern Analysis and Machine Intelligence*.

[20] Ogniewicz R. L., Kübler O. (1995). Hierarchic Voronoi skeletons. *Pattern Recognition*.

[21] Lam L., Suen C. Y. (1992). Thinning methodologies—a comprehensive survey. *IEEE Transactions on Pattern Analysis and Machine Intelligence*.

[22] Bai X., Latecki L. J., Liu W.-Y. (2007). Skeleton pruning by contour partitioning with discrete curve evolution. *IEEE Transactions on Pattern Analysis and Machine Intelligence*.

[23] Siddiqi K., Bouix S., Tannenbaum A., Zucker S. W. Hamilton-Jacobi skeleton.

[24] Grogorishin T., Abdel-Hamid G., Yang Y. (1996). Skeletonization, an electrostatic field-based approach. *Pattern Analysis and Applications*.

[25] Gorelick L., Galun M., Sharon E., Basri R., Brandt A. (2006). Shape representation and classification using the poisson equation. *IEEE Transactions on Pattern Analysis and Machine Intelligence*.

[26] Belongie S., Malik J., Puzicha J. (2002). Shape matching and object recognition using shape contexts. *IEEE Transactions on Pattern Analysis and Machine Intelligence*.

[27] Chaudhuri D., Samal A. (2007). A simple method for fitting of bounding rectangle to closed regions. *Pattern Recognition*.

[28] Ryberg C., Rostrup E., Stegmann M. B. (2007). Clinical significance of corpus callosum atrophy in a mixed elderly population. *Neurobiology of Aging*.

[29] Witelson S. F. (1989). Hand and sex differences in the isthmus and genu of the human corpus callosum. A postmortem morphological study. *Brain*.

[30] Kass M., Witkin A., Terzopoulos D. (1988). Snakes: active contour models. *International Journal of Computer Vision*.

[31] Blum H. (1967). *A transformation for extracting new descriptors of shape*.

[32] Chuang J.-H., Tsai C.-H., Ko M.-C. (2000). Skeletonization of three-dimensional object using generalized potential field. *IEEE Transactions on Pattern Analysis and Machine Intelligence*.

[33] Hu Q., Nowinski W. L. (2003). A rapid algorithm for robust and automatic extraction of the midsagittal plane of the human cerebrum from neuroimages based on local symmetry and outlier removal. *NeuroImage*.

[34] Dempster A. P., Laird N. M., Rubin D. B. (1977). Maximum likelihood from incomplete data via the EM algorithm. *Journal of the Royal Statistical Society: Series B (Statistical Methodology)*.

[35] Direkoglu C., Dahyot R., Manzke M. (2012). On using anisotropic diffusion for skeleton extraction. *International Journal of Computer Vision*.

[36] Weis S., Jellinger K., Wenger E. (1991). Morphometry of the corpus callosum in normal aging and Alzheimer's disease. *Journal of Neural Transmission. Supplementa*.

[37] Hampel H., Teipel S. J., Alexander G. E. (1998). Corpus callosum atrophy is a possible indicator of region- and cell type-specific neuronal degeneration in Alzheimer disease: A magnetic resonance imaging analysis. *JAMA Neurology*.

[38] Hensel A., Wolf H., Kruggel F. (2002). Morphometry of the corpus callosum in patients with questionable and mild dementia. *Journal of Neurology, Neurosurgery & Psychiatry*.

[39] Wu H., Wang D., Shi L., Wen Z., Ming Z. (2014). Midsagittal plane extraction from brain images based on 3D SIFT. *Physics in Medicine and Biology*.

[40] Volkau I., Bhanu Prakash K. N., Ananthasubramaniam A., Aziz A., Nowinski W. L. (2006). Extraction of the midsagittal plane from morphological neuroimages using the Kullback-Leibler's measure. *Medical Image Analysis*.

[41] Liu Y., Collins R. T., Rothfus W. E. (2001). Robust midsagittal plane extraction from normal and pathological 3-D neuroradiology images. *IEEE Transactions on Medical Imaging*.

[42] Adamson C., Beare R., Walterfang M., Seal M. (2014). Software Pipeline for Midsagittal Corpus Callosum Thickness Profile Processing: Automated Segmentation, Manual Editor, Thickness Profile Generator, Group-Wise Statistical Comparison and Results Display. *Neuroinformatics*.

[43] Içer S. (2013). Automatic segmentation of corpus collasum using Gaussian mixture modeling and Fuzzy C means methods. *Computer Methods and Programs in Biomedicine*.

[44] Li Y., Mandal M., Ahmed S. N. Fully automated segmentation of corpus callosum in midsagittal brain MRIs.

[45] Kubicki M., Styner M., Bouix S. (2008). Reduced interhemispheric connectivity in schizophrenia-tractography based segmentation of the corpus callosum. *Schizophrenia Research*.

[46] Cascio C., Styner M., Smith R. G. (2006). Reduced relationship to cortical white matter volume revealed by tractography-based segmentation of the corpus callosum in young children with developmental delay. *The American Journal of Psychiatry*.

[47] Liu I., Chiu C., Chen C., Kuo L., Lo Y., Tseng W. I. (2010). The microstructural integrity of the corpus callosum and associated impulsivity in alcohol dependence: A tractography-based segmentation study using diffusion spectrum imaging. *Psychiatry Research: Neuroimaging*.

[48] Changizi N., Hamarneh G., Ishaq O., Ward A., Tam R. (2010). Extraction of the plane of minimal cross-sectional area of the corpus callosum using template-driven segmentation.. *Medical image computing and computer-assisted intervention : MICCAI ... International Conference on Medical Image Computing and Computer-Assisted Intervention*.

